# Comparison of C-reactive Protein Levels between Serum and Plasma Samples on Long-term Frozen Storage after a 13.8 Year Interval: The JMS Cohort Study

**DOI:** 10.2188/jea.17.120

**Published:** 2007-07-18

**Authors:** Shizukiyo Ishikawa, Kazunori Kayaba, Tadao Gotoh, Yosikazu Nakamura, Kazuomi Kario, Yoshihisa Ito, Eiji Kajii

**Affiliations:** 1 Division of Community and Family Medicine, Center for Community Medicine, Jichi Medical University.; 2 School of Health and Social Services, Saitama Prefectural University.; 3 Department of Public Health, Jichi Medical University.; 4 Division of Cardiology, Department of Internal medicine, Jichi Medical University.; 5 Department of Laboratory Medicine, Asahikawa Medical College

**Keywords:** C-reactive Protein, Stability, Time, Plasma, Japanese

## Abstract

**BACKGROUND:**

C-reactive protein (CRP) is an acute phase reactant, and has been reported as a predictor of cardiovascular diseases. Measurements of high sensitive CRP in thawed samples are possible and the values are thought to remain stable even after frozen storage. However, the long-term stability of CRP values has not been documented. We measured the values of CRP before and after long-term storage, and examined the difference in determined values.

**METHODS:**

High sensitive CRP was measured before and after long-term storage of samples from 99 men and women among the JMS Cohort Study subjects. We selected subjects who underwent measurement of high sensitive CRP at the baseline by stratified sampling methods using baseline CRP values. CRP was measured in serum samples at the baseline and in thawed plasma samples after an average storage period of 13.8 years.

**RESULTS:**

Geometric means of CRP were 0.25 mg/L and 0.59 mg/L before and after storage, respectively. The CRP values were significantly higher after long-term frozen storage than at the baseline (p<0.0001). The both values of logarithm CRP were significantly correlated using Pearson's correlation (r = 0.920, 95% confidence interval: 0.883-0.945).

**CONCLUSION:**

CRP values increased after long-term frozen storage. The CRP values showed a high correlation between before and after long-term storage.

C-reactive protein (CRP) is an acute phase reactant and a marker of inflammation in the human body. Some studies have reported high sensitive CRP (hsCRP) is a marker of cardiovascular diseases not only in western countries^1^^-^^9^ but also in Japan.^10^ Danesh et al^9^ reviewed the relationship between CRP and coronary heart disease (CHD). The odds ratio of the top third to the bottom third was about 1.5 on a meta-analysis.

Because some cross-sectional studies reported that CRP correlate with obesity, high triglycerides, low high-density lipoprotein (HDL) cholesterol and abnormal glucose metabolism, the relationship between CRP and metabolic syndrome has received attention in recent years.^11^^-^^15^ In some other studies, CRP was a predictor of future metabolic syndrome.^16^^-^^19^ Ridker et al^20^ indicated that the interrelationship between hsCRP and metabolic syndrome strongly predicts CHD and CVD death. A recent statement from the Centers for Disease Control and Prevention and the American Heart Association concluded that it is reasonable to measure CRP as an adjunct to the measurement of established risk factors in order to assess the risk of coronary heart disease.^21^

It is necessary to use stored samples in nested-case controlstudies or cohort studies, when we examine new risk factors. Many cohort studies^1^^,^^3^^,^^5^^,^^10^ and nested case-control studies^4^^,^^6^^,^^7^^,^^9^ have examined the relationship between CRP and CVD using frozen samples stored for many years.

Variability in the measurement of CRP under various situations, including freeze/thaw experiments did not affect the levels of CRP over a short period of time.^22^^,^^23^ However, the long-term stability of CRP values in frozen samples has not been examined previously. In the present study, we measured CRP values before and after long-term storage, and examined the long-term variability of CRP values in frozen samples.

## METHODS

HsCRP was measured before and after long-term storage of samples obtained from 99 men and women among the JMS Cohort Study subjects. A detailed description of the JMS Cohort Study was reported previously.^24^ The subjects were population-based and were followed for incidences of stroke and myocardial infarction. CRP was measured with non-thawed serum samples at the baseline in 6,759 participants (2,573 men and 4,186 women). After an average storage period of 13.8 years, we selected 99 samples (45 men and 54 women) by the stratified random sampling method using baseline CRP values. Distribution of CRP values is highly skewed, and we intended to select samples showing various levels of CRP at the baseline.

Blood samples were obtained in the morning after an overnight fast, and drawn from the antecubital vein of seated subjects with minimal tourniquet use. Tubes were centrifuged at 3,000 g for 15 minutes at room temperature within two hours after sampling. After separation, the serum samples were stored at 4 °C in refrigerated containers if analyses were to be performed within two days. Plasma samples were stored in refrigerated containers with dry ice for a maximum of 6 hours, and then frozen as rapidly as possible at −80 °C for storage until laboratory tests were performed.

At the baseline, CRP values were measured from serum samples using highly sensitive nephelometry, a latex particle-enhanced immunoassay (NA Latex CRP Kit, Dade Behring, Tokyo, Japan). The value in the calibrator was assigned using certified reference Material 470 (IRMM, Geel, Belgium), and international plasma protein reference material to achieve international standardization for the assay of CRP. The function of the assay was satisfactory.^25^.The lower limit of detection of CRP in the assay is 0.030 mg/L and undetectable CRP values were recorded as 0.015 mg/L.

We stored frozen plasma samples with trisodium citrate at −80 °C after one freeze/thaw procedure to measure coagulate factors at the baseline, and measured CRP levels using the same methods after an average storage period of 13.8 years. At this time, the assay is sufficiently sensitive to detect 0.050 mg/L of CRP, therefore, undetectable CRP values were recorded as 0.025 mg/L.

Distributions of CRP and triglycerides were skewed, and those were expressed as the geometric means and ± standard deviation (SD). Variables were expressed as mean ± SD except CRP and triglycerides, and categorical data were in proportion. We analyzed the correlation between the two measurements of CRP using Pearson's correlation coefficients with logarithm CRP and 95% confidence interval. T-test was performed between the two measurements. Statistical analysis was performed with SPSS^®^ (Version 14.0J; Chicago, IL, USA).

## RESULTS

**Figure 1.**
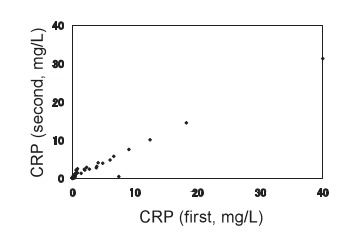
Plots of raw C-reactive protein measurements before and after storage for 13.8 years. Samples were measured at the baseline (CRP, first), and after thawing following long-term frozen storage (CRP, second).

**Figure 2.**
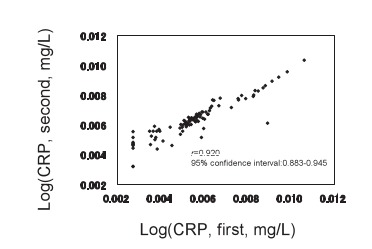
Plots of logarithm of C-reactive protein measurements before and after storage for 13.8 years. Samples were measured at the baseline (CRP, first), and after thawing following long-term frozen storage (CRP, second).

The general characteristics of the subjects were shown in Table 1. The mean and standard deviation of age was 53.6 ± 11.9 years, and those of body mass index were 23.2 ± 3.2 kg/m^2^. Levels of CRP ranged from undetectable to 40.0 mg/L before storage and from undetectable to 31.3 mg/L after storage (Figure 1). Medium levels of CRP were 0.23 mg/L and 0.57 mg/L, and geometric means of CRP were 0.25 mg/L and 0.59 mg/L before and after storage, respectively. The levels of CRP after storage were significantly higher than those before interval (p<0.0001, paired t-test) (Table 2). Distributions of CRP values were similar in both sexes in each measurement, and there was no significant difference in CRP values between men and women at each time point. Correlation was significant between the values of logarithm CRP using Pearson's correlation coefficient (*r* = 0.920, confidence interval: 0.883-0.945) (Figure 2).

**Table 1.** General characteristics of the subjects with two measurements of C-reactive protein

	n	Mean	SD
Age (years)	99	53.6	11.9
Body mas index (kg/m^2^)	81	23.2	3.2
Systolic blood pressure (mmHg)	81	126.0	19.5
Diastolic blood pressure (mmHg)	81	74.9	11.4
Total cholesterol (mg/dL)	99	198.1	34.8
Triglycerides (mg/dL)*	99	110.0	(58.7-206.1)
HDL-cholesterol (mg/dL)	99	46.9	12.2

Sex (Men/Women)	45/54		

Smoking^**^			
Current	16 (20)		
Ex-smoker	15 (19)		
Non-Smoker	50 (62)		

Alcohol drinking^**^			
Current	41 (51)		
Ex-drinker	3 (4)		
Drinker	37 (46)		

* : Geometric mean (± standard deviation) ** : Number (percentage)

**Table 2.** The values of C-reactive protein before and after 13.8 years interval.

	n	Percentale				

		1	25	50	75	99
CRP, first (mg/L)	99	0.015	0.130	0.230	0.470	40
CRP, second (mg/L)	99	0.025	0.307	0.568	0.960	31.3

			Geometric mean		Standard deviation*	
CRP, first (mg/L)	99		0.251		(0.04-1.40)**	
CRP, second (mg/L)	99		0.587		(0.17-1.99)	

CRP, first: measured at the baseline

CRP, second: measured after defrosted with 13.8 years interval

* : Geometric mean ± standard deviation

** : p<0.0001 by paired t-test

## DISCUSSION

We showed the intra-individual variability of CRP values obtained before and after 13.8 years of frozen storage. We found a close relationship between the two measurements, but CRP values examined after long-term storage were significantly higher than those at the baseline.

Our colleagues reported previously that CRP values were lower in Japanese than in western subjects, were less than the lowest detection value (0.03 mg/L) in more than 10% of the subjects in the general population,^26^ and that intra-individual correlation coefficient over 5 years was 0.43.^27^ In the present study, we selected 99 subjects among those who underwent CRP examination at the baseline using the stratified sampling method. Distribution of CRP was highly skewed, and the values of CRP would be mainly in the lower level when random sampling was performed. We tried to examine reproducibility after long-term storage using a wide spectrum of the CRP values; from less than the minimum to more than 30 mg/L.

It has been reported that CRP value remains stable in a frozen sample.^22^^,^^28^ Macy et al^22^ reported several types of intra-individual variability using samples collected at a single time point. They compared sample types; serum, SCAT-1 plasma, SCAT-2 plasma, EDTA plasma, and citrate plasma samples in six individuals, CRP values were lower in sodium citrate tubes than in other types of tubes. They also compared values in a freeze/thaw experiment and thawed samples once, twice, three, and four times using the same 5 types of tubes. There were no significant differences in the mean value of CRP and CRP remained stable throughout multiple freeze/thaw cycles. Aziz et al^23^ confirmed that stability of CRP levels did not differ between serum and plasma samples, or after 7 freeze-thaw cycles. They also found that there were no significant differences in CRP levels with up to a 6 hour delay in specimen processing. Nisson et al^28^ reported in 34 patients admitted to a coronary care unit using 10 year frozen samples that hsCRP showed a predictable slope in the regression between serum and citrate plasma ([hsCRP]citmte=0.902 × [hsCRP]serum − 0.1695). We stored samples at 4°C within 2 hours after processing specimens, after that hsCRP was measured using serum samples within few days. Otherwise, only one thaw-freeze cycle was performed before long-term storage of plasma samples. It was considered that neither our measurement procedure nor storage critically affected the levels of CRP.

We first measured CRP values using serum sample at the baseline, and second using thawed plasma sample after long-term storage. According to the study by Macy et al,^22^ the fact that the CRP values of the second measurement were higher than those of the first was not due to the different types of sample tubes. The variability of differences between the two measurements could not be explained by regression to the mean phenomenon because we selected samples using stratified methods, but only the lower values of CRP at the first measurement showed an increase, while higher CRP levels did not decline. We speculated that differences between the two measurements were caused by evaporation during long-term frozen storage, and especially affected low CRP values. Another possibility is that progressive changes in the pre-cipitability of some proteins might occur. A linear relationship was seen in both Figures 1 and 2, and the values converged toward a high level of CRP.

To our knowledge, this is the first study to examine the stability between before and after long-term interval of frozen storage. Some limitations exist in the present study. First, we selected samples randomly from groups stratified by CRP value at the baseline, not simple random samples. Second, the second measurement was performed after freeze/thaw procedure once at the baseline to measure coagulating factors. Third, we could not confirm the effect of evaporation, because other measurements, such as elctrolyte: potassium, sodium or chloride, were not done. The samples were stored in tempered tubes to prevent evaporation. Nevertheless, evaporation might have occurred during storage, and CRP values in the second measurement might have increased. We could confirm the linear relationship between the two measurements due to stratified selection. We could not estimate how CRP values change during long-term frozen storage, but the results of the present study might characterize the reliability of the long-term storage data. It is considered reasonable to use CRP values of frozen samples in epidemiological studies to estimate risk ratios, such as odds ratio or hazard ratio. However, we should be careful when determining the cut-off level of CRP values as a risk factor based on values obtained using frozen samples after long-term storage.

In conclusion, the CRP values showed a high correlation between before and after long-term storage. In our data, the values after long-term storage were significantly higher than those before storage, although we could not confirm the effect of evaporation. Other studies to examine differences before and after storage for various periods should be conducted in future.
